# Ratio of visceral-to-subcutaneous fat area improves long-term mortality prediction over either measure alone: automated CT-based AI measures with longitudinal follow-up in a large adult cohort

**DOI:** 10.1007/s00261-025-05149-7

**Published:** 2025-08-11

**Authors:** Daniel Liu, Adam J. Kuchnia, Glen M. Blake, Matthew H. Lee, John W Garrett, Perry J. Pickhardt

**Affiliations:** 1 University of Wisconsin–Madison, Madison, USA; 2 School of Biomedical Engineering and Imaging Sciences, King’s College London, London, UK

**Keywords:** Adipose tissue, CT, CT biomarkers, Artificial intelligence, Deep learning

## Abstract

**Background:**

Fully automated AI-based algorithms can quantify adipose tissue on abdominal CT images. The aim of this study was to investigate the clinical value of these biomarkers by determining the association between adipose tissue measures and all-cause mortality.

**Methods:**

This retrospective study included 151,141 patients who underwent abdominal CT for any reason between 2000 and 2021. A validated AI-based algorithm quantified subcutaneous (SAT) and visceral (VAT) adipose tissue cross-sectional area. A visceral-to-subcutaneous adipose tissue area ratio (VSR) was calculated. Clinical data (age at the time of CT, sex, date of death, date of last contact) was obtained from a database search of the electronic health record. Hazard ratios (HR) and Kaplan–Meier curves assessed the relationship between adipose tissue measures and mortality. The endpoint of interest was all-cause mortality, with additional subgroup analysis including age and gender.

**Results:**

138,169 patients were included in the final analysis. Higher VSR was associated with increased mortality; this association was strongest in younger women (highest compared to lowest risk quartile HR 3.32 in 18-39y). Lower SAT was associated with increased mortality regardless of sex or age group (HR up to 1.63 in 18-39y). Higher VAT was associated with increased mortality in younger age groups, with the trend weakening and reversing with age; this association was stronger in women.

**Conclusion:**

AI-based CT measures of SAT, VAT, and VSR are predictive of mortality, with VSR being the highest performing fat area biomarker overall. These metrics tended to perform better for women and younger patients. Incorporating AI tools can augment patient assessment and management, improving outcome.

## Introduction

Obesity is a well-established risk factor for several adverse clinical outcomes including cardiometabolic disease and mortality [1, 2]. While BMI and waist circumference are traditionally utilized clinical measures, they are relatively crude measures that fail to capture crucial aspects of adiposity [3, 4]. Notably, there is an important distinction between visceral adipose tissue (VAT) and subcutaneous adipose tissue (SAT). VAT is known to be metabolically active while SAT, although associated with known metabolic implications, is thought to have protective benefits as well. [4, 5], [6].

Artificial intelligence (AI) provides a novel avenue to measure adipose tissue, which was previously difficult to obtain. While CT is considered the gold standard for quantifying adipose tissue, traditional manual methods are somewhat time consuming [6, 7]. Fully automated AI-based CT tools can derive accurate, objective measures of SAT and VAT that can be used clinically to optimize care without high overhead [8–12]. The most pragmatic approach involves taking a cross-sectional area sample at the level of L3, which serves as a representative measure of a patient’s total abdominal adipose tissue volume [13].

VAT and SAT area, obtained through this L3 sampling technique, were shown to be associated with clinical outcomes [14–17]. However, distribution of adipose tissue between these compartments, captured by the visceral-to-subcutaneous adipose tissue area ratio (VSR), likewise has been found to have important clinical implications separate from VAT or SAT ratio in isolation [18]. This clinical insight is likely attributable to unique properties of VAT and SAT [13].

Although the clinical implications of these three adipose tissue area-based measures are well documented, it is unclear which is the best and how correlated the predictive value is to the adipose tissue measure. Likewise, more data is needed to ascertain how the predictive value of these measures differ demographically. Therefore, the purpose of this study was 1) to assess the performance of adipose tissue area in predicting all-cause mortality in a large cohort of adult patients, 2) determine whether adipose tissue area alone or a ratio of VAT to SAT is a better predictor of survival, and 3) establish demographic age and sex differences for these three measures.

## Methods

### Patient cohort

**Fig. 1:**
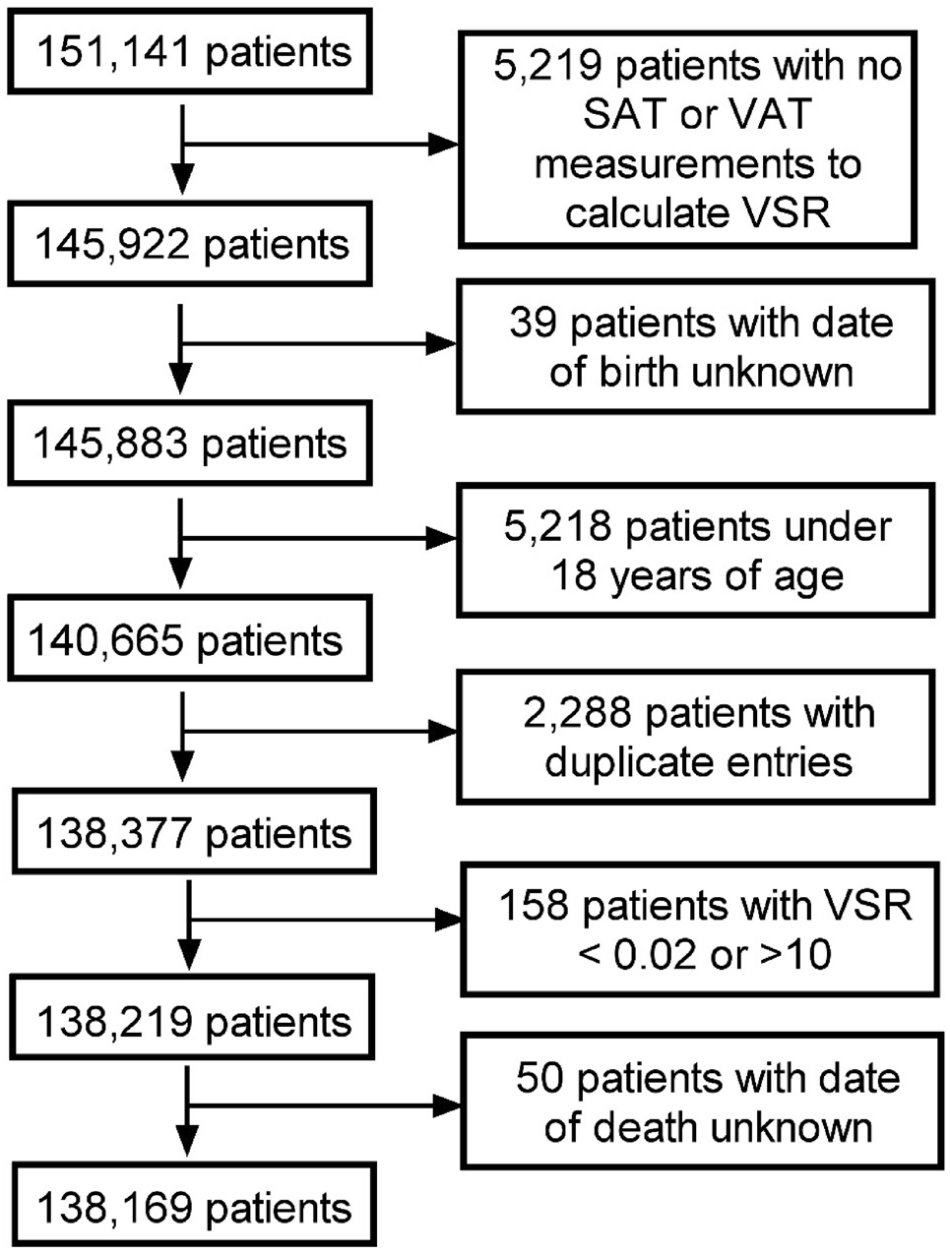
Flowchart depicting patient selection and exclusion

This retrospective cohort study is HIPAA-compliant and was approved by the institutional review board at the University of Wisconsin School of Medicine and Public Health. A total of 151,141 patients who underwent abdominal CT scans between 2000 and 2021 at the University of Wisconsin Hospitals and Clinics were included in this study. Patients were only included once, and the earliest available CT image was used for analysis. A detailed algorithm of the electronic health record (EHR) was utilized to identify key clinical information. Patients were excluded for tool failure, extreme outlier biomarker values, age younger than 18 years, or missing EHR data. This process is depicted as a flowchart in Fig. 1. The cohort was further subdivided by age (18–39, 40–59, 60–79, and 80 + years) as well as gender (male and female, self-reported) to evaluate the role these played on survival indices.

### CT technique

Any CT that captured the full abdomen (L1-L5) was included in this study. A variety of CT scanning protocols were included to maximize generalizability, resulting in varying contrast phases, x-ray tube potentials, and dose ranges. Scanners were of various models from different manufacturers including GE HealthCare, Siemens Healthineers, and Philips Healthcare.

### Fully automated artificial intelligence based abdominal adipose tissue segmentation

The automated artificial intelligence-based adipose tissue segmentation pipeline utilized in this study is a research tool that has been previously described and validated [9, 13, 19, 20]. The current pipeline methodology was summarized in a recent publication [21]. The pipeline is robust and generalizable to CT vendor and technique [19, 22–24]. Briefly, the tool works by preparing the CT data for processing followed by adipose tissue segmentation. Preprocessing involves ensuring correct patient orientation in standard Left-Anterior–Superior voxel ordering, reconstructing the series to contiguous 3-mm slices using a standard soft-tissue kernel, and CT number normalization by removing vendor Hounsfield unit (HU) offset. Then, vertebral body localization is performed using a convolutional neural network (CNN) based unsupervised body part regression algorithm. This results in predicted level labeling for the slice numbers corresponding to the approximate midpoint of vertebral bodies T12 through L5.

Once the CT data are prepared, adipose tissue segmentation and quantification is performed. This process consisted of five general steps and was done on the slice corresponding to the midaxial plane of L3. (1) A body mask was created with a region-growing algorithm, segmenting low-intensity pixels outside the body and removing the CT Table (2) Noise reduction was performed using an anisotropic diffusion filter. (3) Voxels between -274 HU and -49 HU were labeled adipose tissue. (4) An active contour modeling algorithm created body contours, modifying them across multiple iterations. This resulted in an external contour around the outside of the body and an internal contour around the abdominal wall musculature. (5) Adipose tissue is quantified. SAT was defined as any voxel labeled as adipose tissue between the external and internal contours. VAT was defined as any voxel labeled as adipose tissue within the internal contour. For VAT and SAT area, voxel area was totaled to calculate the cross-sectional area (mm^2^). The VSR was calculated by taking the ratio of VAT area to SAT area.

**Fig. 2:**
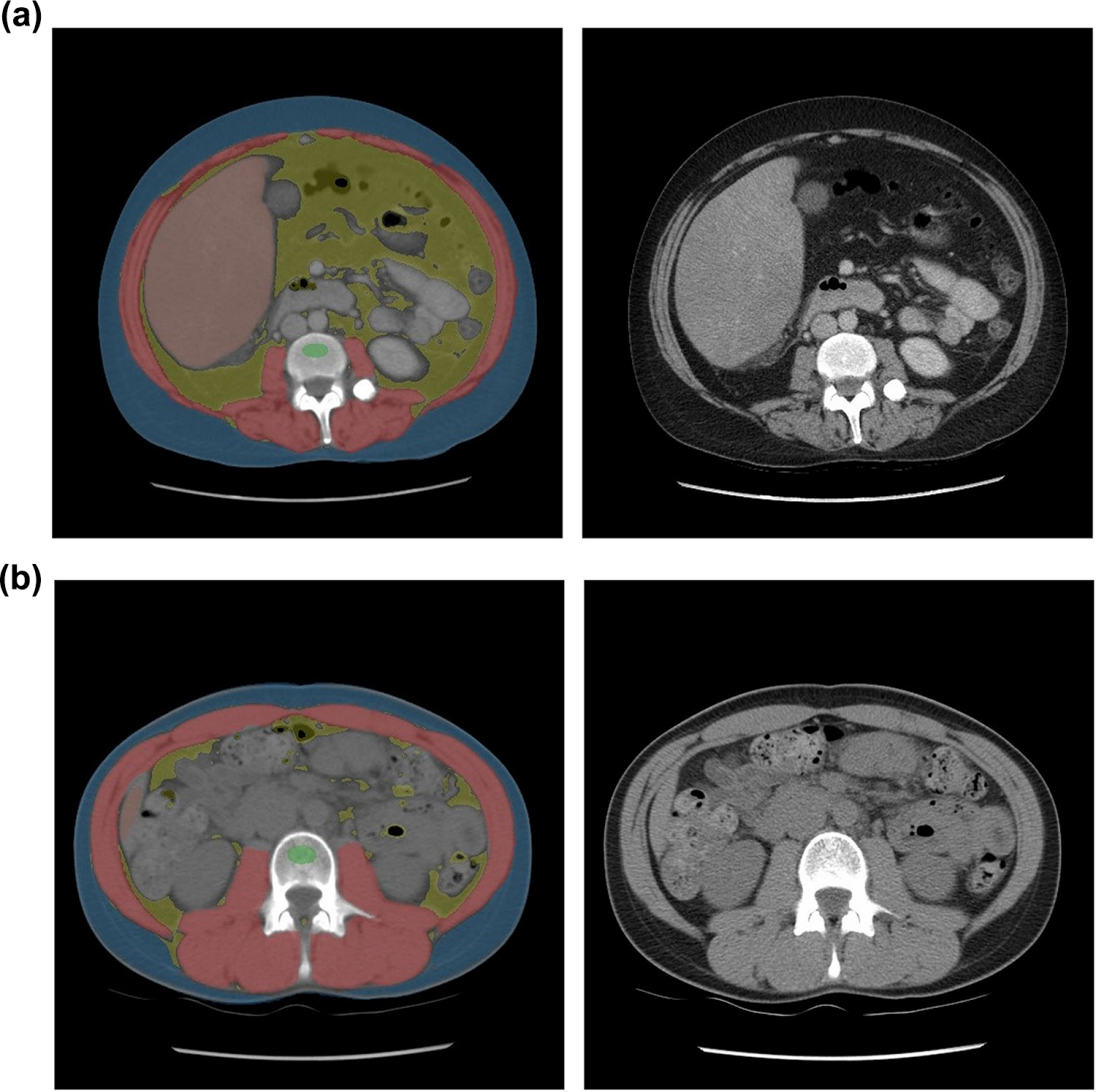
Examples of segmented CT images at the L3 level. **A** 33-year-old woman presenting with vomiting, bloody diarrhea, and abdominal distention and underwent contrast-enhanced abdominal CT. VAT is segmented with gold and SAT is segmented with blue (additional pipeline segmentation of muscle, liver, and trabecular bone is also shown). At the time of CT, she was found to have a VAT area of 198.6 cm^2^, SAT area of 187.3 cm^2^, and VSR of 1.06. She presented again roughly 3 months later after suffering cardiac arrest and passed away due to brain injury secondary to the cardiac arrest. **B** 26-year-old woman presenting with left sided abdominal pain and underwent non-contrast abdominal CT to evaluate for kidney stones. VAT is segmented with gold and SAT is segmented with blue. At the time of CT, she was found to have a VAT area of 23.4 cm^2^, SAT area of 71.5 cm^2^, and VSR of 0.33. She was last seen nearly 20 years later at a routine office visit

The adipose tissue segmentation results for every CT image are depicted on an automatically generated, color-coded quality assurance image which can be visually inspected for accuracy of segmentation (Fig. 2).

### Clinical outcomes

Clinical variables used in this study were obtained from the EHR. Key patient data included patient date of birth and sex. Key clinical outcomes included date of death and date of last contact. Date of last contact was based on the most recent documented in-person clinical encounter, such as an office visit or lab draw. Patients with a date of death were included in the deceased cohort, while those without were included in the alive cohort with date of last contact being used to calculate the survival time.

### Statistical analysis

A cox proportional hazard model was utilized to assess the relationship between the time to mortality and the adipose tissue predictor variables. Hazard ratios (HR) were computed for each adipose tissue measure. For the whole cohort, patients were divided into 12 groups by percentile and a HR calculated for each subgroup. This was also done for groups split by sex and age group, with the patients split into quartiles (Q1-Q4). For time-to-event survival analysis, Kaplan–Meier (KM) curves were created for VSR, again divided by sex and age group with patients further split into quartiles (Q1-Q4). Statistical analysis was performed using Statistics Kingdom software (Statistics Kingdom, Melbourne, Australia). A p-value < 0.05 was considered statistically significant.

## Results

### Patient sample

There were 138,169 patients included in this study (mean age 53 years, 52% female), of which, 90% were Caucasian. The median clinical follow-up time was 5.6 years (interquartile range, 1.9 to 11.1 years). 28,392 out of the 138,169 patients died over the course of clinical follow-up. Patient characteristics and summary data are summarized in Table 1.

**Table 1:** Patient characteristics

	Men					Women				
Age Group	N	Median Age (IQR) (y)	Median SAT Area (IQR) (cm^2^)	Median VAT Area (IQR) (cm^2^)	Median VSR (IQR)	N	Median Age (IQR) (y)	Median SAT Area (IQR) (cm^2^)	Median VAT Area (IQR) (cm^2^)	Median VSR (IQR)
All ages	66,310	55 (41–66)	170.1 (115.3–241.5)	192.4 (96.3–295.9)	1.01 (0.65–1.48)	71,859	53 (39–65)	229.6 (145.1–335.8)	96.4 (42.2–174.9)	0.40 (0.25–0.62)
Age18-39	15,663	29 (24–35)	144.6 (78.7–233.3)	91.0 (37.2–176.6)	0.61 (0.40–0.88)	18,415	29.0 (24–35)	208.6 (122.0–337.8)	47.2 (23.0–104.0)	0.24 (0.16–0.36)
Age 40–59	24,880	51 (46–55)	177.6 (123.6–253.0)	200.9 (114.6–294.5)	1.01 (0.71–1.42)	27,787	50 (46–55)	245.8 (159.5–353.7)	102.6 (48.0–179.5)	0.40 (0.26–0.59)
Age 60–79	21,875	67 (63–72)	177.9 (129.2–241.2)	248.1 (152.7- 348.6)	1.29 (0.90–1.77)	21,034	67 (63–72)	235.2 (159.0–330.7)	133.2 (70.7–213.1)	0.54 (0.37–0.78)
Age 80 plus	3892	83.5 (81–87)	152.2 (111.4–202.2)	236.3 (148.8–331.1)	1.49 (1.07–1.97)	4623	84 (82–88)	181.8 (114.3–254.8)	117.0 (64.4–187.2)	0.65 (0.45–0.92)

*SAT* Subcutaneous adipose tissue, *VAT* visceral adipose tissue, *VSR* Visceral-to-subcutaneous area ratio, *IQR* Interquartile range

### Adipose tissue measures

**Fig. 3:**
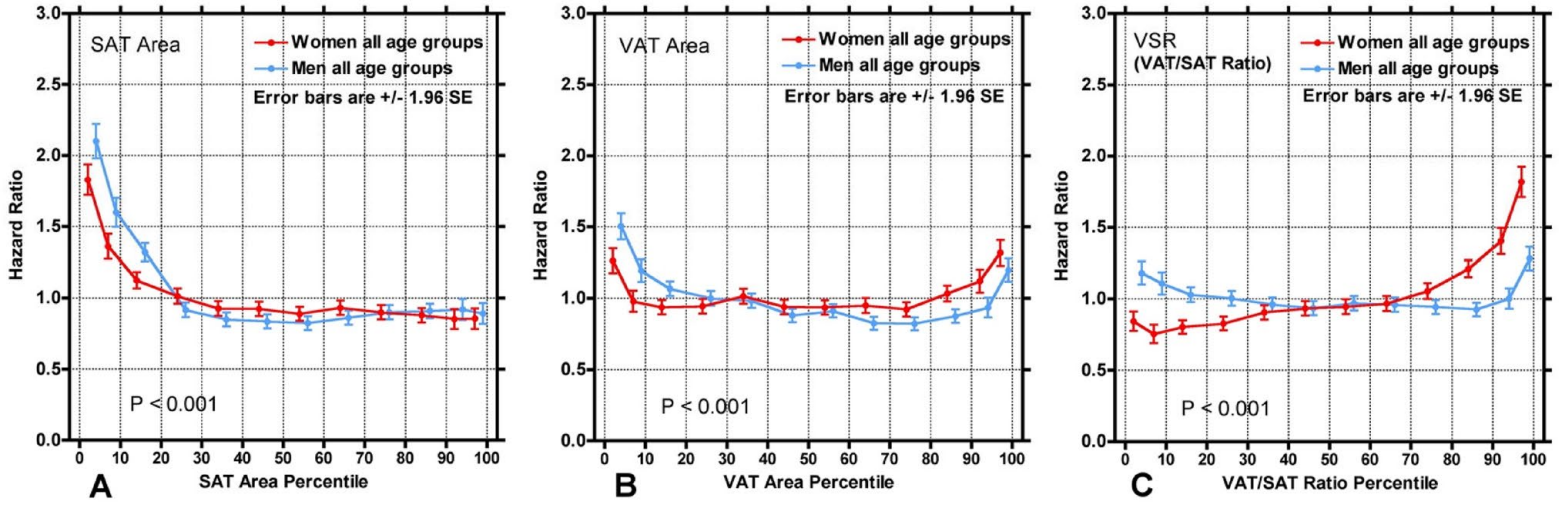
Hazard ratio as a function of (**A**) SAT area, **B** VAT area, and **C** VSR. Points are plotted every 5 percentiles between the 0 to 10th percentiles and 90th to 100th percentiles (i.e., 0–5%, 5–10%, 90–95%, 95–100%) and every 10 percentiles between the 10th to 90th percentiles. The points are slightly offset on the x-axis to prevent overlapping and obscuring. Notably, lower SAT area, lower and higher VAT area, and higher VSR was associated with an increased hazard ratio for all-cause mortality. P-values show the statistical comparison between the twelve percentile points for men and women using the chi-squared test. *SAT* Subcutaneous adipose tissue, *VAT* Visceral adipose tissue, *VSR* Visceral-to-subcutaneous area ratio, *SE* Standard error

**Fig. 4:**
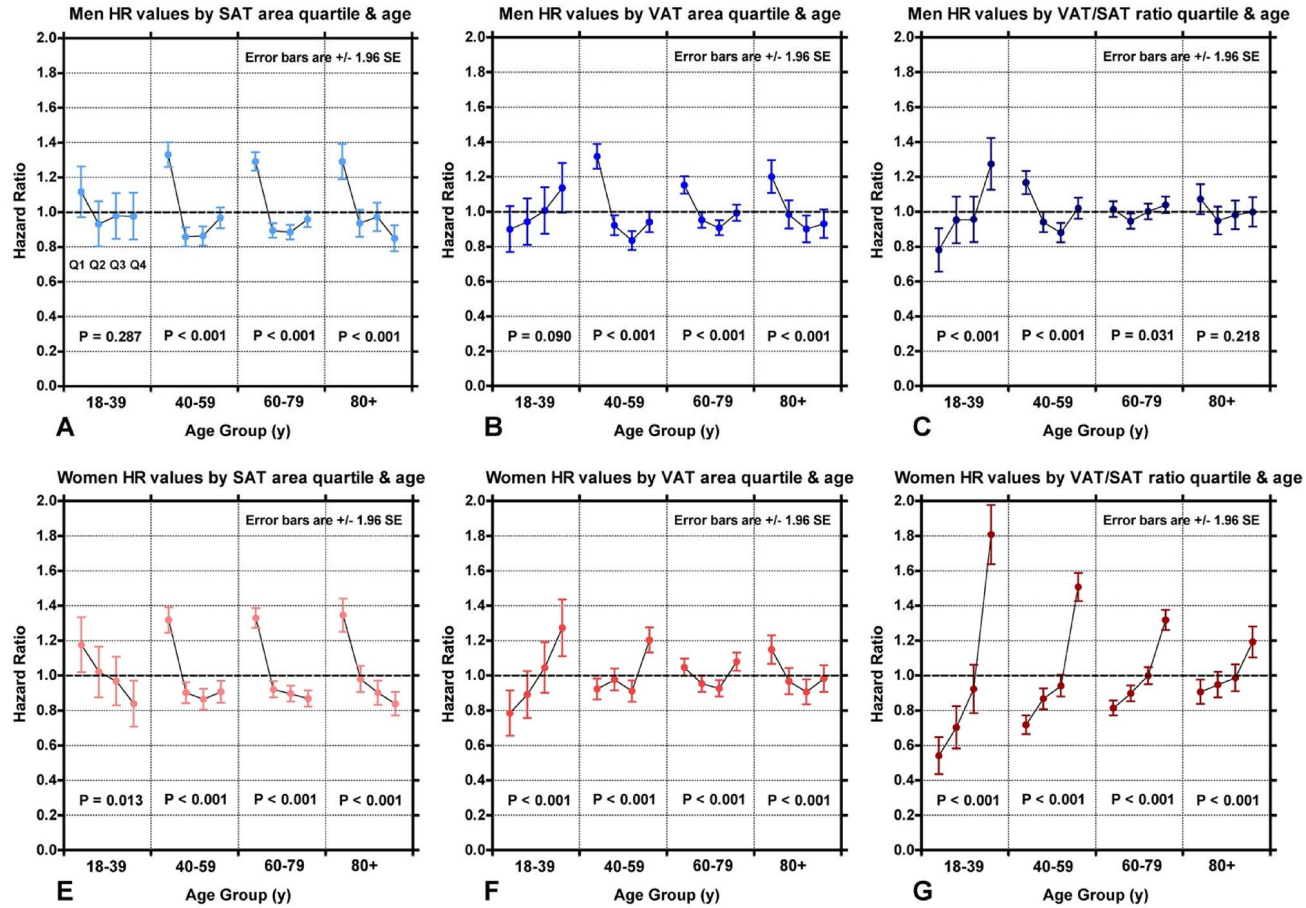
The hazard ratios as a function of the four quartiles (Q1, Q2, Q3, Q4) of the adipose tissue measurements. The subjects are separated by age and gender (Men **A**-**C**; Women **D**-**F**). P-values show the statistical comparison between the four quartiles in each age group using the chi-squared test. *SAT* Subcutaneous adipose tissue, *VAT* Visceral adipose tissue, *VSR* Visceral-to-subcutaneous area ratio, *SE* Standard error. Lower SAT area had an increased HR for all-cause mortality for most age groups (**A** and **D**). For VAT, higher VAT area and an increased HR for younger women, with the trend weakening and eventually reversing with age (**E**); lower VAT area had a higher HR in men (**B**). For VSR, high VSR had an increased HR across all age groups for women with the greatest increase in the youngest cohort (**F**); men likewise had an increased HR with high VSR in younger cohorts, but that association is minimal in older cohorts (**C**)

Hazard ratios for all-cause mortality as a function of SAT area, VAT area, and VSR are depicted in Fig. 3. Of note, there was an increased HR for mortality for those in the lowest percentiles of SAT area and VAT area. Similarly, there was an increased HR for those in the highest percentiles of VAT area. Conversely, there was no change or a slightly decreased HR for those in the highest percentiles of SAT area. For VSR, there was an increased HR for the highest percentiles, with a slight decrease observed in lower percentiles. When stratified by age group, low SAT area was associated with increased mortality for the majority (Fig. 4). Low VAT area was associated with an increased HR for older men, whereas for women, increased VAT area was associated with an increased HR in the younger cohorts while that trend reverses in older cohorts, with lower VAT area being associated with increased risk of mortality. High VSR was associated with increased mortality in women of all age cohorts, whereas for men, high VSR only had a higher risk of mortality in the youngest cohort.

Table 2 depicts the HRs comparing highest risk to lowest risk quartiles of a given adipose tissue measure for the same age groups in Fig. 4. VSR generally had the largest HRs, up to 3.32 (2.58–4.06) in 18- to 39-year-old women and 1.64 (1.32–1.96) in 18- to 39-year-old men. SAT area had the highest HR of 1.55 (1.42–1.68) in 40 to 59-year-old men and 1.62 (1.44–1.80) in women over 80 years old. VAT area had the highest HR of 1.58 (1.45–71) in 40–59-year-old men and 1.63 (1.29–1.97) in 18- to 39-year-old women.

**Table 2:** Hazard ratios expressed as the ratio of the hazard ratios of the highest and lowest risk quartiles in Fig. 4

Sex	Age Group	SAT Area HR (95% CI)	VAT Area HR (95% CI)	VSR HR (95% CI)
Men	Age18-39	1.20 (0.97–1.43)	1.27 (1.03–1.51)	1.64 (1.32–1.96)
	Age 40–59	1.55 (1.42–1.68)	1.58 (1.45–1.71)	1.33 (1.22–1.44)
	Age 60–79	1.47 (1.37–1.57)	1.27 (1.19–1.35)	1.10 (1.03–1.17)
	Age 80 plus	1.53 (1.35–1.71)	1.34 (1.18–1.50)	1.14 (1.01–1.27)
Women	Age18-39	1.42 (1.13–1.71)	1.63 (1.29–1.97)	3.32 (2.58–4.06)
	Age 40–59	1.52 (1.39–1.65)	1.32 (1.20–1.44)	2.10 (1.91–2.29)
	Age 60–79	1.53 (1.42–1.64)	1.17 (1.09–1.25)	1.62 (1.51–1.73)
	Age 80 plus	1.62 (1.44–1.80)	1.27 (1.14–1.40)	1.34 (1.20–1.48)

*HR* Hazard ratio; 95% CI: 95% confidence interval *SAT* Subcutaneous adipose tissue, *VAT* Visceral adipose tissue, *VSR* Visceral-to-subcutaneous area ratio

**Fig. 5:**
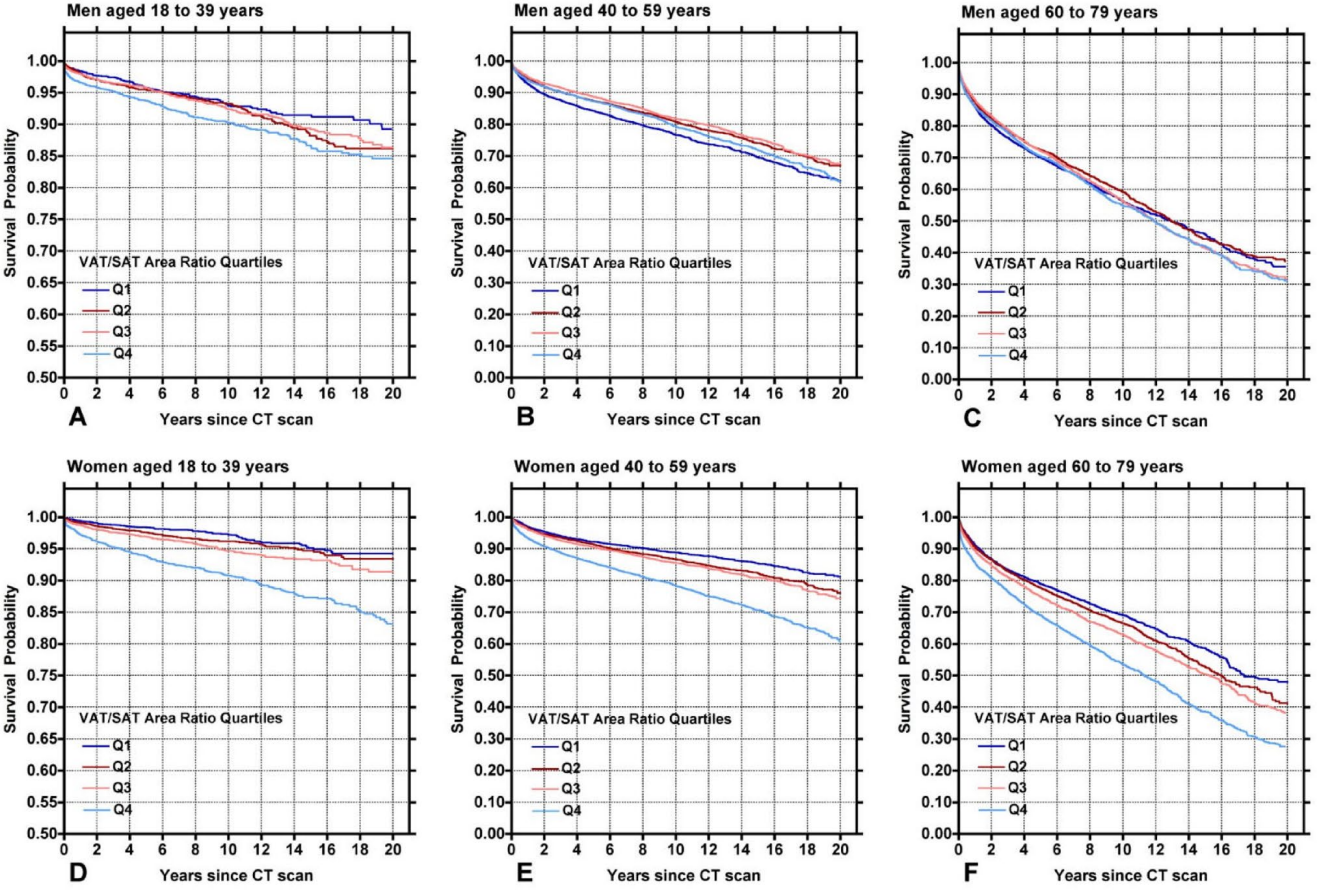
Kaplan–Meier Curves for survival based on VSR, separated by age and gender (Men **A**-**C**; Women **D**-**F**). Women with high VSR had the most notable drop off in survival, especially in the 18–39 years cohort (**D**) and the 40–59 years cohort (**E**)

KM curves depicting time to mortality from date of CT are shown in Fig. 5, separated by quartiles. Visual survival curve separation was most pronounced in women across age cohorts, with the greatest separation being in the highest quartile (Q4) in the 18–39 years and 40–59 years cohorts. There was notably less separation for men of all age groups.

## Discussion

In this study, adipose tissue area was measured using fully automated AI-based CT tools, and the predictive value of these measures for all-cause mortality was assessed. Overall, we found that each of the adipose tissue area measures were predictive of all-cause mortality and that VSR demonstrated better survival prediction overall compared with VAT or SAT area alone, with exceptions. More specifically, increased risk of mortality was seen for patients with low SAT area, high and low VAT area, and high VSR. These trends differed slightly when broken down by gender and divided into four age cohorts. For men, increased mortality was seen in those with low SAT area, high VAT area but reversing to low VAT area with older age, and high VSR in younger patients. For women, increased mortality was seen in those with low SAT area, high VAT area in younger patients but again reversing with advancing age, and high VSR. In general, adipose tissue area measures had a U-shaped curve for all-cause mortality, where patients on the extremes of both ends saw an increased risk. These trends tended to be stronger in female patients. High VAT area was typically associated with increased risk of all-cause mortality in younger patients, but the relationship was weaker and even reversed in the older cohorts. The association between high VSR and increased risk of all-cause mortality tended to persist throughout all age cohorts. Regardless, VSR generally outperformed VAT area and SAT area alone.

The relationship between obesity and mortality is well documented [25]. Our study found increased VAT but not SAT to be predictive of increased mortality, with stronger association in younger patients and women. VAT has more endocrine activity, particularly in pathways that contribute to numerous metabolic pathologies [5, 26]. Moreover, high VAT volume was shown to be strongly associated with the risk of developing cardiometabolic disease [27]. Thus, increased visceral adiposity plays a more significant role in the pathogenesis of numerous diseases that can adversely impact survival, which fits the trend observed in this study. On the other hand, the data for SAT volume is less straightforward. High SAT area has protective benefits in some cases such as hip fractures and cancer [14, 28]. Likewise, SAT was found to have a neutral effect on developing cardiometabolic disease, and even in cases where there was an association it was weaker than VAT [27]. However, other studies found an marked increase in cardiovascular events and mortality for those with high SAT area [29]. These contrasting relationships could explain why there was no increased mortality risk with high SAT area, and at higher levels even a decreased risk.

Conversely, we also found increased mortality risk in the lowest percentiles of VAT and SAT. Cachexia is also known to increase mortality risk, and these body composition changes can be quantified on CT [30]. Likewise, anorexia also presents a mortality risk [31, 32]. Although both low VAT and SAT area could suggest these conditions, studies show low SAT area to have superior prognostic value in certain conditions such as cancer [28, 33]. This could explain the stronger hazard ratios in the lowest percentile for SAT area compared to similar percentiles for VAT [33]. In all, metabolic conditions resulting in low adipose tissue volume can negatively impact survival, potentially even more significantly than high volume.

Although adipose tissue area alone had predictive value, the area ratio (VSR) had even better performance in this study. Previous studies show VSR to likewise be a strong predictor of adverse outcome, including numerous metabolic conditions such as cardiovascular disease and type 2 diabetes [18, 29, 34–36]. Similar to this study, VSR has been shown to outperform isolated volume and area measures, suggesting VSR captures information not represented by adipose tissue area alone [4, 37, 38]. Understanding how both VAT and SAT contribute to a patient’s overall adiposity is crucial for patient care. In many clinical cases, VSR is a superior biomarker and therefore should be considered. These AI-based CT tools are an efficient way to obtain this critical clinical information and can add significantly to more common measures such as patient weight or BMI.

This study also broke down how these relationships differ demographically. Notably, high VSR and VAT area measures had a stronger association with all-cause mortality in women, agreeing with previously published results. [13, 37] Prior studies showed differences in adipose tissue deposition, with an “apple shape” body habitus for men and “pear shape” body habitus for women [13, 39]. Likewise, men typically have higher amounts of VAT than women [39]. Therefore, a VAT increase in women may be more detrimental to overall health as it constitutes a greater percent increase in their total adipose tissue volume. This suggests that VAT area and VSR may be a better metric for female patients.

Age also impacts the relationship between adipose tissue area and mortality, especially for VAT. A prior study showed that age modified the relationship between visceral adiposity and all-cause mortality [40]. More specifically, they used a sex-specific visceral adiposity index (VAI) and found that high VAI was associated with increased mortality only in patients under 60 years of age [40]. Similarly, another study found high VAT area to be associated with increased all-cause mortality in patients < 65 years, a relationship that weakens or reverses with age [41]. The results of this study found similar trends of increased mortality risk in younger cohorts with high VAT or VSR that weakens or reverses in older cohorts. High VAT is associated with chronic conditions like diabetes and hypertension, which typically decrease long-term survival, meaning older patients may not live long enough to see a notable decrease in overall survival [42]. Furthermore, adipose tissue redistributes with age. Age-related changes in particular result in increased VAT but decreased SAT, independent of changes in total adiposity, body weight, or waist circumference [43]. Therefore, although an older patient may have more VAT, it is not drastically greater relative to the rest of the population, meaning there will be a less than expected impact on survival. Disease-specific relationships could further reinforce this trend. Cancer tends to be more prevalent in older populations, and cachexia is a poor prognostic sign for these patients [28]. However, in some cancer cohorts, higher VAT is associated with better survival probabilities [44]. It is possible older patients are more reliant on their reserves, which outweighs any potential long-term adverse impact of high adipose tissue volume. Notably, it is possible cancer results in death before any cardiometabolic conditions develop. This combined effect could blunt the effect of increased VAT, while the cachexia effect persists, resulting in the increased mortality for low VAT but unchanged mortality for high VAT seen in the older cohorts of this study.

There is growing evidence that supports the importance of discerning how VAT and SAT contribute to overall adiposity as it provides insight that cannot be gleaned from cruder measures like BMI or waist circumference. Segmenting adipose tissue through CT is one avenue to accomplishing this and potentially the most ideal method given its accuracy and high prevalence in clinical practice [7]. Incorporating these approaches and tools into practice is crucial to improving patient care. Beyond optimizing treatment of disease, these measures can also be used in a preventative fashion. For example, adipose tissue measures can be combined with other body composition measures from the same abdominal CT image into an explainable model that is predictive of future outcome [14–17]. Subsequently, preventative measures can be taken based on which measures are abnormal.

We acknowledge limitations in this study. This was a single-center retrospective study. Given the CT was obtained for any indication including those already with underlying disease, this cohort may be more biased toward certain outcomes compared to the general population. Additionally, this cohort included both contrast and non-contrast exams. Although previous studies have established correction factors and examined whether the CT type impacts outcome, it may be advantageous to have separate protocols for these two types of CT exams. As mentioned above, the adipose tissue areas used in this study were taken from a single slice at the level of L3. This means the measures are simply a sample of a patient’s abdominal adipose tissue and in some cases could result in over- or underestimating the actual adipose tissue volume. However, given the state of AI technology and the costs, segmenting the entire abdominal adipose tissue can be less practical.

In conclusion, fully automated AI-based body composition tools can derive accurate measures of VAT and SAT area from abdominal CT images. High VAT, low VAT, and low SAT area were predictive of all-cause mortality. The VSR combines VAT and SAT area into an all-in-one metric, and high VSR was both predictive of all-cause mortality and outperformed adipose tissue area alone. All adipose measures were stronger predictors of all-cause mortality in women and younger patients. Implementing these tools and quantifying adipose tissue can augment patient care and improve clinical outcome by identifying additional risk factors with data that were previously unutilized.

## Data Availability

The complete processing pipeline used for this study is not currently available under an open-source license and is not currently commercially available. However, the code can be made available to academic researchers upon reasonable request to the corresponding author, for non-commercial use and subject to institutional and legal agreements.

## References

[1] Mokdad AH, Ford ES, Bowman BA, et al. Prevalence of Obesity, Diabetes, and Obesity-Related Health Risk Factors, 2001. JAMA 2003;289(1):76-79. 10.1001/jama.289.1.76. 12503980 10.1001/jama.289.1.76

[2] Kaess BM, Jozwiak J, Mastej M, et al. Association between anthropometric obesity measures and coronary artery disease: a cross-sectional survey of 16 657 subjects from 444 Polish cities. Heart 2010;96(2):131-135. 10.1136/hrt.2009.171520. 19651624 10.1136/hrt.2009.171520

[3] Prentice AM, Jebb SA. Beyond body mass index. Obesity Reviews 2001;2(3):141-147. 10.1046/j.1467-789x.2001.00031.x. 12120099 10.1046/j.1467-789x.2001.00031.x

[4] Kaess BM, Pedley A, Massaro JM, Murabito J, Hoffmann U, Fox CS. The ratio of visceral to subcutaneous fat, a metric of body fat distribution, is a unique correlate of cardiometabolic risk. Diabetologia 2012;55(10):2622–30. (In eng). 10.1007/s00125-012-2639-5. 10.1007/s00125-012-2639-5 PMC3636065 22898763

[5] Ibrahim MM. Subcutaneous and visceral adipose tissue: structural and functional differences. Obesity Reviews 2010;11(1):11-18. 10.1111/j.1467-789X.2009.00623.x. 19656312 10.1111/j.1467-789X.2009.00623.x

[6] Shuster A, Patlas M, Pinthus JH, Mourtzakis M. The clinical importance of visceral adiposity: a critical review of methods for visceral adipose tissue analysis. British Journal of Radiology 2014;85(1009):1-10. 10.1259/bjr/38447238. 10.1259/bjr/38447238 PMC3473928 21937614

[7] Park HJ, Shin Y, Park J, et al. Development and Validation of a Deep Learning System for Segmentation of Abdominal Muscle and Fat on Computed Tomography. Korean J Radiol 2020;21(1):88–100. (10.3348/kjr.2019.0470). 10.3348/kjr.2019.0470 PMC6960305 31920032

[8] Pickhardt PJ. Value-added Opportunistic CT Screening: State of the Art. Radiology 2022;303(2):241–254. (In eng). 10.1148/radiol.211561. 10.1148/radiol.211561 PMC9083232 35289661

[9] Lee SJ, Liu J, Yao J, Kanarek A, Summers RM, Pickhardt PJ. Fully automated segmentation and quantification of visceral and subcutaneous fat at abdominal CT: application to a longitudinal adult screening cohort. Br J Radiol 2018;91(1089):20170968. (In eng). 10.1259/bjr.20170968. 10.1259/bjr.20170968 PMC6223139 29557216

[10] Pickhardt PJ, Summers RM, Garrett JW, et al. Opportunistic Screening: Radiology Scientific Expert Panel. Radiology 2023;307(5):e222044. (In eng). 10.1148/radiol.222044. 10.1148/radiol.222044 PMC10315516 37219444

[11] Pickhardt PJ, Graffy PM, Perez AA, Lubner MG, Elton DC, Summers RM. Opportunistic Screening at Abdominal CT: Use of Automated Body Composition Biomarkers for Added Cardiometabolic Value. Radiographics 2021;41(2):524–542. (In eng). 10.1148/rg.2021200056. 10.1148/rg.2021200056 PMC7924410 33646902

[12] Pickhardt PJ, Graffy PM, Zea R, et al. Automated CT biomarkers for opportunistic prediction of future cardiovascular events and mortality in an asymptomatic screening population: a retrospective cohort study. Lancet Digit Health 2020;2(4):E192-E200. (Article) (In English). 10.1016/s2589-7500(20)30025-x. 10.1016/S2589-7500(20)30025-X PMC7454161 32864598

[13] Liu D, Garrett JW, Lee MH, Zea R, Summers RM, Pickhardt PJ. Fully automated CT-based adiposity assessment: comparison of the L1 and L3 vertebral levels for opportunistic prediction. Abdom Radiol (NY) 2023;48(2):787-795. 10.1007/s00261-022-03728-6. 36369528 10.1007/s00261-022-03728-6

[14] Liu D, Garrett JW, Perez AA, et al. Fully automated CT imaging biomarkers for opportunistic prediction of future hip fractures. Br J Radiol 2024;97(1156):770-778. 10.1093/bjr/tqae041. 38379423 10.1093/bjr/tqae041 PMC11027263

[15] Liu D, Binkley NC, Perez A, et al. CT image-based biomarkers acquired by AI-based algorithms for the opportunistic prediction of falls. BJR Open 2023;5(1):20230014. (In eng). 10.1259/bjro.20230014. 10.1259/bjro.20230014 PMC10636337 37953870

[16] Liu D, Ji D, Garrett JW, et al. Automated abdominal CT imaging biomarkers and clinical frailty measures associated with postoperative deceased-donor liver transplant outcomes. European Radiology 2025. 10.1007/s00330-025-11523-2. 40121592 10.1007/s00330-025-11523-2

[17] Pickhardt PJ, Kattan MW, Lee MH, et al. Biological age model using explainable automated CT-based cardiometabolic biomarkers for phenotypic prediction of longevity. Nature Communications 2025;16(1):1432. 10.1038/s41467-025-56741-w. 39920106 10.1038/s41467-025-56741-w PMC11806064

[18] Ladeiras-Lopes R, Sampaio F, Bettencourt N, et al. The Ratio Between Visceral and Subcutaneous Abdominal Fat Assessed by Computed Tomography Is an Independent Predictor of Mortality and Cardiac Events. Revista Española de Cardiología (English Edition) 2017;70(5):331-337. 10.1016/j.rec.2016.09.010. 10.1016/j.rec.2016.09.010 27765543

[19] Lee MH, Liu D, Garrett JW, et al. Comparing fully automated AI body composition measures derived from thin and thick slice CT image data. Abdominal radiology (New York) 2024;49(3):985–996. (In eng). 10.1007/s00261-023-04135-1. 10.1007/s00261-023-04135-1 38158424

[20] Yan K, Lu L, Summers R. Unsupervised Body Part Regression via Spatially Self-ordering Convolutional Neural Network. 2018 (http://arxiv.org/abs/1707.03891).

[21] Garrett JW, Pickhardt PJ, Summers RM. Methodology for a fully automated pipeline of AI-based body composition tools for abdominal CT. Abdominal Radiology 2025. 10.1007/s00261-025-04951-7. 40293521 10.1007/s00261-025-04951-7 PMC12568892

[22] Toia GV, Garret JW, Rose SD, Szczykutowicz TP, Pickhardt PJ. Comparing fully automated AI body composition biomarkers at differing virtual monoenergetic levels using dual-energy CT. Abdominal radiology (New York) 2024 (In eng). 10.1007/s00261-024-04733-7. 10.1007/s00261-024-04733-7 39643734

[23] Moeller AR, Garrett JW, Summers RM, Pickhardt PJ. Adjusting for the effect of IV contrast on automated CT body composition measures during the portal venous phase. Abdominal radiology (New York) 2024;49(7):2543–2551. (In eng). 10.1007/s00261-024-04376-8. 10.1007/s00261-024-04376-8 38744704

[24] Pooler BD, Garrett JW, Southard AM, Summers RM, Pickhardt PJ. Technical Adequacy of Fully Automated Artificial Intelligence Body Composition Tools: Assessment in a Heterogeneous Sample of External CT Examinations. AJR American journal of roentgenology 2023;221(1):124–134. (In eng). 10.2214/ajr.22.28745. 10.2214/AJR.22.28745 37095663

[25] Brown JC, Harhay MO, Harhay MN. Anthropometrically-predicted visceral adipose tissue and mortality among men and women in the third national health and nutrition examination survey (NHANES III). American Journal of Human Biology 2017;29(1):e22898. 10.1002/ajhb.22898. 10.1002/ajhb.22898 PMC5241265 27427402

[26] Kershaw EE, Flier JS. Adipose Tissue as an Endocrine Organ. The Journal of Clinical Endocrinology & Metabolism 2004;89(6):2548-2556. 10.1210/jc.2004-0395. 15181022 10.1210/jc.2004-0395

[27] Sam S. Differential effect of subcutaneous abdominal and visceral adipose tissue on cardiometabolic risk. Hormone Molecular Biology and Clinical Investigation 2018; 33(1). 10.1515/hmbci-2018-0014. 10.1515/hmbci-2018-0014 29522417

[28] Ebadi M, Martin L, Ghosh S, et al. Subcutaneous adiposity is an independent predictor of mortality in cancer patients. British Journal of Cancer 2017;117(1):148-155. 10.1038/bjc.2017.149. 28588319 10.1038/bjc.2017.149 PMC5520211

[29] Lee MH, Zea R, Garrett JW, Summers RM, Pickhardt PJ. AI-based abdominal CT measurements of orthotopic and ectopic fat predict mortality and cardiometabolic disease risk in adults. European Radiology 2025;35(1):520-531. 10.1007/s00330-024-10935-w. 38995381 10.1007/s00330-024-10935-w

[30] Agustsson T, Wikrantz P, Rydén M, Brismar T, Isaksson B. Adipose tissue volume is decreased in recently diagnosed cancer patients with cachexia. Nutrition 2012;28(9):851-855. 10.1016/j.nut.2011.11.026. 22480800 10.1016/j.nut.2011.11.026

[31] Korndörfer SR, Lucas AR, Suman VJ, Crowson CS, Krahn LE, Melton LJ. Long-term Survival of Patients With Anorexia Nervosa: A Population-Based Study in Rochester, Minn. Mayo Clinic Proceedings 2003;78(3):278-284. 10.4065/78.3.278. 12630579 10.4065/78.3.278

[32] Arcelus J, Mitchell AJ, Wales J, Nielsen S. Mortality Rates in Patients With Anorexia Nervosa and Other Eating Disorders: A Meta-analysis of 36 Studies. Archives of General Psychiatry 2011;68(7):724-731. 10.1001/archgenpsychiatry.2011.74. 21727255 10.1001/archgenpsychiatry.2011.74

[33] Han J, Tang M, Lu C, Shen L, She J, Wu G. Subcutaneous, but not visceral, adipose tissue as a marker for prognosis in gastric cancer patients with cachexia. Clinical Nutrition 2021;40(9):5156-5161. 10.1016/j.clnu.2021.08.003. 34461589 10.1016/j.clnu.2021.08.003

[34] Kim EH, Kim H-K, Lee MJ, et al. Sex Differences of Visceral Fat Area and Visceral-to-Subcutaneous Fat Ratio for the Risk of Incident Type 2 Diabetes Mellitus. dmj 2021;46(3):486–498. 10.4093/dmj.2021.0095. 10.4093/dmj.2021.0095 PMC9171158 34911174

[35] Edwards LA, Bugaresti JM, Buchholz AC. Visceral adipose tissue and the ratio of visceral to subcutaneous adipose tissue are greater in adults with than in those without spinal cord injury, despite matching waist circumferences. The American Journal of Clinical Nutrition 2008;87(3):600-607. 10.1093/ajcn/87.3.600. 18326597 10.1093/ajcn/87.3.600

[36] Pisitsak C, Lee JGH, Boyd JH, Coxson HO, Russell JA, Walley KR. Increased Ratio of Visceral to Subcutaneous Adipose Tissue in Septic Patients Is Associated With Adverse Outcome*. Critical Care Medicine 2016;44(11):1966-1973. 10.1097/ccm.0000000000001870. 27513541 10.1097/CCM.0000000000001870

[37] He H, Ni Y, Chen J, et al. Sex difference in cardiometabolic risk profile and adiponectin expression in subjects with visceral fat obesity. Transl Res 2010;155(2):71–7. (In eng). 10.1016/j.trsl.2009.08.003. 10.1016/j.trsl.2009.08.003 20129487

[38] Yeoh AJ, Pedley A, Rosenquist KJ, Hoffmann U, Fox CS. The Association Between Subcutaneous Fat Density and the Propensity to Store Fat Viscerally. The Journal of Clinical Endocrinology & Metabolism 2015;100(8):E1056-E1064. 10.1210/jc.2014-4032. 26062015 10.1210/jc.2014-4032 PMC4525002

[39] Sun J, Xu B, Lee J, Freeland-Graves JH. Novel Body Shape Descriptors for Abdominal Adiposity Prediction Using Magnetic Resonance Images and Stereovision Body Images. Obesity (Silver Spring) 2017;25(10):1795-1801. 10.1002/oby.21957. 28842953 10.1002/oby.21957

[40] Sun Q, Wang S, Han X, et al. The association between visceral adiposity index and long-term all-cause mortality shows age-related disparities: a nationwide cohort study. BMC Public Health 2025;25(1):1266. 10.1186/s12889-025-22428-6. 40181244 10.1186/s12889-025-22428-6 PMC11969692

[41] Saad RK, Ghezzawi M, Horanieh R, et al. Abdominal Visceral Adipose Tissue and All-Cause Mortality: A Systematic Review. Frontiers in Endocrinology 2022; Volume 13 - 2022 (Systematic Review) (In English). 10.3389/fendo.2022.922931. 10.3389/fendo.2022.922931 PMC9446237 36082075

[42] Xu C, Zhang P, Cao Z. Cardiovascular health and healthy longevity in people with and without cardiometabolic disease: A prospective cohort study. eClinicalMedicine 2022;45. 10.1016/j.eclinm.2022.101329. 10.1016/j.eclinm.2022.101329 PMC8904213 35284807

[43] Kuk JL, Saunders TJ, Davidson LE, Ross R. Age-related changes in total and regional fat distribution. Ageing Res Rev 2009;8(4):339-48. 10.1016/j.arr.2009.06.001. 19576300 10.1016/j.arr.2009.06.001

[44] Taemkaew K, Churungsuk C, Khanungwanitkul K, Keeratichananont W, Tanutit P, Liabsuetrakul T. Body Composition As Prognostic Markers For Survival In Non-Metastatic Non-Small Cell Lung Cancer. Clinical Nutrition ESPEN 2023;54:504-505. 10.1016/j.clnesp.2022.09.141.

